# The prevalence and patterns of hearing loss in Jordan: A cross-sectional study

**DOI:** 10.1371/journal.pone.0304125

**Published:** 2024-08-15

**Authors:** Safa Alqudah, Margaret Zuriekat, Saja Hassan, Heba Mahafdeh, Zainab Alqudah, Aya Shatarah, Ghufran Smadi

**Affiliations:** 1 Faculty of Applied Medical Sciences, Department of Rehabilitation Sciences, Associate Professor of Audiology, Ph.D., Jordan University of Science and Technology, Irbid, Jordan; 2 Department of Special Surgery, School of Medicine, Assistant Professor of Audiology, Ph.D., The University of Jordan & Jordan University Hospital, Amman, Jordan; 3 Department of Physiology and Neurobiology, Surgical Neurophysiologist, CNIM, Master of Science, University of Connecticut, Storrs, CT, United States of America; 4 Faculty of Applied Medical Sciences, Department of Rehabilitation Sciences, Speech and Hearing Technician, Bachelor in Speech and Hearing, Jordan University of Science and Technology, Irbid, Jordan; 5 Faculty of Applied Medical Sciences, Department of Allied Medical Sciences, Assistant Professor of Audiology, Ph.D., Jordan University of Science and Technology, Irbid, Jordan; 6 Faculty of Applied Medical Sciences, Department of Rehabilitation Sciences, Speech and Hearing Technician, Bachelor in Speech and Hearing, Jordan University of Science and Technology, Irbid, Jordan; 7 Faculty of Applied Medical Sciences, Department of Rehabilitation Sciences, Speech and Hearing Technician, Bachelor in Speech and Hearing, Jordan University of Science and Technology, Irbid, Jordan; UFPE: Universidade Federal de Pernambuco, BRAZIL

## Abstract

**Background:**

Hearing loss is known to be a serious issue that impedes human communication. The World Health Organization (WHO) estimates that approximately 20 in 100,000 newborns demonstrate congenital hearing impairments, leading to severely impacted language, academic, and social abilities of these children.

**Objective:**

The reduced quality of life and work productivity among hearing-impaired individuals eventually affects societal outcomes and development. Since limited studies address the nature of hearing-impaired individuals in Jordan, this research aimed to define the prevalence and nature of hearing loss in Jordan, highlighting important facts about hearing loss epidemiology across Jordanians.

**Methods:**

The current research focused on assessing hearing function for 1000 individuals over 12 years to define the rate, most prominent configurations, and the most common characteristics of hearing difficulties in Jordan.

**Results:**

The results showed that sixty-three per 1,000 people have hearing loss, most frequently sensorineural hearing loss. The age range of people with hearing loss was 12 to 89 years old, with a median age of 51. The incidence of hearing loss appeared at a later age (33.33%, X^2^ = 15.74, *p*<0.05). The percentages of hearing loss were similar across the main Jordanian governorates (X^2^ = 7.14, *p*>0.05), with sensorineural hearing loss reported to be the most common type of hearing loss (N = 46, 73.00%), and mild is the most frequent severity (N = 25, X^2^ = 23.58, *p*<0.05). No statistical variation was reported in the prevalence of hearing loss based on gender.

**Conclusion:**

The rate of hearing loss in Jordan is higher than worldwide prevalence, which was assumed to be due to genetic factors impacting the auditory system. These findings will assist in creating effective hearing conservation programs to reasonably prevent or minimize the spread of hearing loss in Jordan.

## Introduction

Hearing loss is known to be a serious issue that impedes human communication [1]. The World Health Organization (WHO) estimates that approximately 20 in 100,000 newborns demonstrate developmental hearing impairments, severely impacting language, academic, and social abilities [2]. The reduced quality of life and work productivity among deaf and hard-of-hearing individuals eventually affects societal outcomes and development [2].

Hearing loss is any problem in the hearing process that prevents individuals from hearing correctly. This could be due to impaired auditory sensitivity and diminished speech intelligibility. Individuals with hearing loss are sometimes described as deaf or hard of hearing based on the type, degree, and configuration of hearing impairment [3]. The impairment in hearing function is reported to be one of the primary causes of communication disorders [3]. Studies have shown that hearing deficit is the most insidious developmental disease occurring among infants immediately after birth [4]. Congenital hearing loss is approximately 20 per 100,000 births, exceeding other defects that occur directly after childbearing, such as low intelligence, cleft lip and palate, Down syndrome, limb defects, spina bifida, sickle cell anemia, and phenylketonuria [5]. Being born with hearing loss can cause developmental delay in children, making it difficult for them and their caregivers to cope [1]. Most hearing complaints are reported to be acquired throughout the lifetime. According to previous literature, ear infections are the first cause of hearing loss among school-aged children [6]. Moreover, one in three Americans over 65 years old suffers from minor to severe hearing troubles that develop due to aging [7], known as presbycusis [8]. Nearly 30 million Americans listen to damaging, loud sounds in their work environments daily [9], which draws the attention of government agencies, healthcare providers, and administrators of public and private sectors to support the application of occupational noise protection programs.

Other studies from Middle Eastern countries, Egypt and Oman, have shown that conductive hearing loss and presbycusis, defined as sensorineural hearing loss in older people, occur at the highest recurrent percentage [10,11]. The increased prevalence of hearing loss is an uprising problem, which the WHO presented with the estimation of disabling hearing loss could rise to 630 million by 2030 and may be over 900 million by 2050 (9.6%). There are likely to be rapid increases for South Asia, East Asia, and Sub-Saharan Africa. The growth in Asia Pacific, Latin America, and the Caribbean is relatively slower but still substantial. The high-income region, Middle East, and North Africa are slightly slower again, and finally, comparatively slower growth rates for Central/Eastern Europe and Central Asia [12].

Unfortunately, limited literature discusses the prevalence of hearing loss among the Jordanian population. Two studies illustrated that the rate of neonatal hearing loss among Jordanian infants is approximately 15/1000 [13,14], which is more widespread than that in other nations, including the United States (1.45/1000), Belgium (3.18/1000), Turkey (3.6/1000), the UEA (1.8/1000), and Oman (1.2/1000) [15–19]. In addition, a study demonstrated that one-quarter of noise-exposed industrial workers in Jordan have NIHL. Age >35 years, high noise level, exposure for more than ten years, and not using hearing protective devices were significantly associated with an increased risk of NIHL [20]. Another study on the prevalence and risk factors for hearing loss among infants in Jordan demonstrated that of the 63042 infants examined, 966 (1.5%) were confirmed to have significant hearing loss [14].

Knowing more about the profile of hearing loss in Jordan is crucial for highlighting the nature and basis of congenital and acquired hearing loss epidemiology across Jordanians. Thus, it will increase the awareness of healthcare providers and the public about the required conservational procedures, reducing the incidence of hearing deterioration controlled by Jordanian society’s genetic and environmental factors. This will be beneficial in assessing the need to provide audiological services to the population, covering them through insurance companies, and increasing the spotlight on the current hearing services in Jordan.

Previous literature has not discussed the pattern of hearing loss in the Jordanian population in terms of the most common type, severity, laterality, and configuration of hearing loss and which gender and geographical location are most affected by hearing issues. Since studies dealing with the nature of hearing loss in Jordanians are limited, this research aimed to determine the prevalence and pattern of hearing loss in Jordan. Therefore, it will highlight important facts about the epidemiology of hearing loss among Jordanians. The prevalence of hearing loss in the Jordanian population was estimated in this study by performing audiological procedures on 1,000 subjects participating from different community facilities across the country. Additionally, further investigation was conducted on the widespread nature of hearing loss among Jordanians, considering the highly prevalent types, degrees, and configurations of the studied impairment. Such statistics should be vital to the national health organizations responsible for controlling the increased demand for healthcare providers to offer high-quality public services within a limited budget in developing countries. Consequently, studying the common risk factors for hearing impairment specific to the Jordanian population will help minimize the prevalence of this disorder efficiently and select appropriate treatment options. In addition, the incidence of any disease could be dramatically reduced by raising public awareness about its issues. A study stated that the available data on NIHL and attitudes toward hearing loss and hearing protection among university students are limited, indicating little awareness in Jordanian society [21]. The WHO recommends that healthcare providers develop conservation programs targeting a specific community to manage major hearing concerns such as pediatric hearing loss, impacted wax, infected ears, and overexposure to harmful noises [22]. Determining the trend of hearing loss in Jordan will provide an excellent opportunity to focus the necessary protection acts on the most severe hearing complications that impact Jordanian society.

## Methodology

This study was designed on a cross-sectional basis to conduct comprehensive hearing assessments on 1,000 people from different regions in Jordan after randomly selecting them from 5,000 Jordanians who had completed the hearing screening; that sample size was chosen to achieve a 95% confidence interval, 80% power for the study, and a 5% error margin. The data included females and males older than 12 years, those with normal or impaired hearing function (developmental or acquired), and hearing aid and cochlear implant users; children younger than 12 years were excluded due to their auditory system immaturity. Auditory immaturity is known for the difficulty of segregating tested tones from masking noises while the hearing thresholds are in the adult population [23]. The study protocol was approved by the Institutional Research Board (IRB) of Jordan University of Science and Technology (27/119/2018).

### Sampling method

The study was held in the period between 2018 and 2020 using a stratified, multistage systematic sampling method based on a similar published protocol [24,25]. For this purpose, a sample of relevant size was drawn from the population despite enumerating each unit of the population [25]. In the current research, a combination of stratified and systematic sampling was preferred. In the case of systematic sampling, the value of k was calculated by dividing the population size by the sample size. After obtaining k, each unit from the population, known as a factor of k, was included in the sample until the required sample size was obtained [26]. Moreover, the study sampling scheme was executed in three stages. In the first stage, the sample included five strata representing the largest governorates. It is known that Jordan has five geological provisions and 12 administrative governorates. The geography of Jordan has been divided into five layers involving (a) Limestone in the interior desserts and the highlands, (b) Sandstone ridges in the Jordanian rift margins and Wadi Rum area, (c) Primeval basement rocks, (d) Basalt desert in the northeast (e) The Jordan rift Valley [27], which were covered in the sample of the study. In terms of administrative division, Jordan is composed of twelve governorates. These governorates are distributed in three regions: Irbid, Ajloun, Jerash, and Mafraq governorates in the northern region; the governorates of Balqa, Amman, Zarqa, and Madaba in the central region; the governorates of Karak, Tafilah, Ma’an, and Aqaba in the southern region. The sample includes at least two or three of the most important governorates in the northern and center regions. In the second stage, a systematic sampling technique was utilized to determine an individual from each area. Every fifth individual within the chosen sample was enumerated and included in the model [28]. In the last stage, all the selected individuals were asked to sign written consent forms to agree to participate in the study. Obtaining consent from parents or guardians of the minors included in the study was ensured to be achieved. Since the individuals were assigned from each region, the study did not involve any chances of bias in selecting study participants. Out of the 5,000 subjects eligible to participate in the study, only 1000 members underwent the hearing measurement. The first stage, collecting 5000 individuals, was selected from 19 health centers and hospitals across the targeted provinces. Then, 1000 were randomly selected from the 5000 and underwent complete diagnostic procedures at King Abdullah University Hospital. The exclusion rate of the study was calculated to be 301 out of 5301 (6%).

### Hearing screening and evaluation

During the first stage of this research, effective hearing screenings were run on all subjects using an AA222 Middle Ear Analyzer and Audiometer to separate those with a high probability of hearing impairment from apparently well-hearing participants; the applied screening protocol followed the American Speech–Language–Hearing Association (ASHA) guidelines, which recommend that before the hearing screening, case history and otoscopic examination are preferable to be done [29]. Then, pure tone air conduction was performed at 25 dB (HL) for 1000, 2000, and 4000 Hz in each ear; the subject should respond to stimuli at 25 dB (HL) at all targeting frequencies in both ears to pass the screening.

The second stage involved conducting complete diagnostic hearing evaluations on individuals who failed the hearing screening and were suspected of having an auditory injury. Hearing evaluations were completed at the Speech and Hearing Clinic in King Abdullah University Hospital, where all equipment was calibrated every six months during the data collection phase. To address the risk factors for hearing loss in Jordan, before each assessment, subjects were asked to provide a case history, including information about demographic characteristics, general medical status, hearing status, family and social history, symptoms/health conditions associated with hearing loss, medication history, and noise and occupational history.

Eight qualified and well-experienced assistants administered the screening and diagnostic audiological procedures. The diagnostic battery included examining the subject’s hearing capability by otoscopic examination, tympanometry, and air and bone conduction audiometry. The hearing thresholds were determined at the most important frequencies for speech (250, 500, 1000, 2000, 4000, and 8000 Hz) generated by a pure tone audiometer. Two inserted earphone transducers delivered these signals to the subjects’ ears. The sound level was decreased in 10 dB steps and increased in 5 dB increments, from 30 dB (HL) to the threshold. If a clear response occurs, the level is decreased by 10 dB. If no response was observed, the tone would be presented at 50 dB HL, then at successive increments of 10 dB until a response is reached [29]. Thresholds were recorded for every tested frequency. The hearing thresholds were defined as the lowest stimulus levels to which the subject responded. Bone conduction thresholds were obtained to identify the type of hearing loss, if present, at 500, 1000, 2000, and 4000 Hz. Pure tone audiometry was performed inside the audiological booth and administered as specified by the American Speech-Language-Hearing Association guidelines. To categorize participants into the normal or impaired hearing groups, hearing loss was reported when the pure tone average of thresholds at 500, 1000, and 2000 Hz exceeded 25 dB in one or both ears. The degree of hearing loss was classified as mild (26–40 dB (HL)), moderate (41–55 dB (HL)), moderately severe (56–70 dB (HL)), severe (71–90 dB (HL)), and profound (>90 dB (HL)) [29]. Since hearing damage can affect different parts of the auditory system, the hearing loss detected in study participants was additionally classified as conductive, sensorineural, and mixed based on observing the air-bone gap [29].

### Statistical analysis

In this research study, data were collected from 1000 individuals selected from Jordan, 503 males (50.3%) and 497 females (49.7%). All participants who failed the screening at 25 dB (HL) for 1000, 2000, and 4000 Hz were comprehensively examined for hearing loss at additional frequencies, 250, 500, 1000, 2000, 4000, and 8000 Hz. A participant was reported to have impaired hearing if the average of pure tone thresholds at 500, 1000, and 2000 Hz was recognized as higher than 25 dB (HL) in one or both ears. Univariate analysis was performed using Statistical Package of Social Sciences (SPSS) version 22 to calculate the frequencies, crude rates, and estimated proportion of the hearing loss. The chi-squared test was used to specify the hearing loss patterns that are significantly dominant in Jordan.

## Results

The Kolmogorov‒Smirnov and Shapiro‒Wilk tests were considered for testing the normality of the data. The results indicated that the data are nonparametric, with *p-values* lower than 0.05 in both tests. Additionally, the visual inspection of normality, such as histograms with fitted normal curves and Q-Q plots, confirmed the numerical findings.

### Demographic characteristics

A sample of five thousand people was collected during the initial recruitment process. The sample was then reduced to include only the target population, namely “k” units. “k” units were defined as the population nominated by sample collectors after verifying their suitability to participate in the study and were coded with the number 5, representing the number of targeted governorates, and its amplifications. The study was double-blind, as neither potential participants nor examiners knew which respondents would be included in the final set of data and statistical analysis. Following the sampling process, two hundred people from each governorate were qualified for the following research stages.

The findings indicated that the prevalence of hearing loss in the Jordanian population was 0.06%, which is sixty-three out of every thousand persons. The average age of those with hearing loss was 51 years old, and those with hearing loss ranged from 12 to 89 years old.

Males comprised most hearing-impaired population (N = 37, 58.70%), while the rest were females (N = 26, 41.30%). Their professional status varied: thirty-one out of fifty-nine people were not employed (N = 31, 49.21%), healthcare providers (N = 3, 4.76%), teachers (N = 4, 6.35%), engineers (N = 3, 4.76%), managers (N = 2, 3.17%), fieldworkers (N = 3, 4.76%), secretaries (N = 2, 3.17%), retirees (N = 8, 12.69%), or other (N = 3, 4.76%). Four people could not be reached for this characteristic. Moreover, regarding education status, bachelor’s degrees made up the bulk of subjects’ educational backgrounds (N = 19, 30.16%), and regarding their marital status, the majority were married (N = 40, 63.49%; Table 1).

**Table 1:** Demographic characteristics of 63 individuals with hearing loss.

Variable	Frequency	Percentage
**Gender**		
Male	37	58.70%
Female	26	41.30%
**Profession**		
Not employed	31	49.21%
Health-care provider	3	4.76%
Teacher	4	6.35%
Engineer	3	4.76%
Manager	2	3.17%
Fieldworker	3	4.76%
Secretary	2	3.17%
Retired	8	12.69%
Other	3	4.76%
Missing	4	6.35%
**Education**		
Elementary	14	22.22%
Intermediate	8	12.69%
High School	10	15.87%
Diploma	2	3.17%
Bachelor	19	30.16%
Master	4	6.35%
Missing	6	9.52%
**Material status**		
Single	14	22.22%
Married	40	63.49%
Widow	2	3.17%
Missing	7	11.11%

### Prevalence of hearing loss in Jordan

The prevalence of hearing loss was determined to be sixty-three out of one thousand participants, with the other participants having normal hearing. The nonparametric chi-squared test was used to compare the prevalence of hearing loss across the governorates in Jordan. The percentages of hearing loss were not significantly different among the governorates (X2 = 7.14, *p*>0.05; Table 2).

**Table 2:** The prevalence of hearing loss in Jordan.

Governorate	Frequency (1000)	Percentage	*P-* value	Statistics
Jerash	18	1.8%	>0.05	7.71a
Zarqa	15	1.5%		
Amman	14	1.40%		
Irbid	11	1.10%		
Ajloun	5	0.05%		

The results revealed that there was a significant difference in the onset age of hearing loss (X^2^ = 15.74, *p*<0.05), with early age falling between 12 and 30 years old (N = 559/1000, 55.9%), middle age falling between 31 and 65 years old (N = 390/1000, 39.00%), and late age falling over 65 years old (N = 51/1000, 5.1%); most cases of hearing loss occurred at a late age (N = 17, 33.33%) followed by middle age (N = 33, 0.08%), and early age (N = 13, 0.02%). Additionally, the hearing loss risk of each sex was compared, suggesting no substantial variations between the female and male participants (X^2^ = 1.92, p >0.05; Table 3).

**Table 3:** The prevalence of hearing loss associated with age and gender.

Age category	Frequency (63/1000)	Percentage	*P-* value	Statistics
Late age	17/51	33.33%	<0.05	15.74
Middle age	33/390	0.08%		
Early age	13/559	0.02%		
**Gender**	**Frequency (63/1000)**	**Percentage**	***P-* value**	**Statistics**
Male	37/503	7.36%	>0.05	1.92
Female	26/497	5.23%		

### The pattern of hearing loss in Jordan

The chi-squared test was performed to compare the onset, severity, and type of hearing loss in Jordan.

There was a significant difference in the severity of hearing loss (X^2^ = 23.58, *p*<0.05), ranging from mild (N = 25, 39.68%), moderate (N = 13, 20.63%), moderately severe (N = 14, 22.22%), severe (N = 10, 15.78%), and to profound (N = 1, 1.59%). A significant variation in the type of hearing loss was noticed (X^2^ = 45.24, *p* <0.05). In Jordan, sensorineural hearing loss was shown to be the most common type of hearing loss (N = 46, 73.00%), followed by mixed hearing loss (N = 11, 17.50%) and conductive hearing loss (N = 6, 9.50%). Different configurations of hearing loss were reported: flat (thresholds of tested frequencies did not differ more than 20 dB from each other; N = 17, 27.00%), sloping (thresholds appeared at equal or successively higher levels as frequency value increased; N = 10, 15.90%), tent-shaped (one or more adjacent thresholds between 500 and 4000 Hz were ≤ 20 dB compared to the better threshold at 250 or 8000 Hz; N = 10, 15.90%), reverse sloping (thresholds reported at equal or successively lower levels from low to high frequencies; N = 6, 9.50%), high frequency (Thresholds at low frequencies are relatively normal with hearing loss is mainly localized at high frequencies; N = 10, 15.90%), notch (threshold occurring between 3000 and 6000 Hz compared to better thresholds at lower and higher frequencies; N = 4, 6.30%), cookie bite shape (one or more adjacent thresholds between 500 and 4000 Hz were ≤ 20 dB compared to the poorer threshold at 250 or 8000 Hz N = 3, 4.80%) and corner (small amount of residual hearing in the low frequencies but no recordable threshold on the rest of the audiogram; N = 3, 4.80%; X^2^ = 20.68, *p* <0.05; Table 3).

The chi-squared test was used to compare hearing loss laterality, bilateral or unilateral. It was found that bilateral hearing loss (N = 52, 82.50%) was significantly higher than unilateral hearing loss (N = 11, 17.50%; X^2^ = 26.68, *p* <0.05; Table 4).

**Table 4:** The pattern of hearing loss in Jordan.

Pattern	Frequency (63)	Percentage	*P-* value	Statistics
		Hearing Loss Severity		
Mild	25	39.68%		
Moderate	13	20.63%		
Moderately Severe	14	22.22%	<0.05	23.58
Severe	10	15.87%		
Profound	1	1.59%		
		**Hearing Loss Type**		
Sensorineural hearing loss	46	73.00%		
Mixed hearing loss	11	17.50%	<0.05	45.24
Conductive hearing loss	6	9.50%		
**Hearing Loss Configuration**				
Flat	17	27.00%		
Sloping	10	15.90%	<0.05	20.68
Tent shaped	10	15.90%		
Reverse sloping	6	9.50%		
High frequency	10	15.90%		
Notch	4	6.30%		
Cookie bite shape	3	4.80%		
Corner	3	4.80%		
		**Laterality**		
Bilateral hearing loss	52	82.50%	<0.05	26.68
Unilateral hearing loss	11	17.50%		

## Discussion

In this study, the prevalence of hearing loss among the Jordanian population was 0.06%. The present study suggested that Jordan has a lower prevalence of hearing loss than many other nations, with 16.5% in the United States [30], 10.2% in England [31], 16.2% in Germany [32], and 16.02% in Egypt [10]. Meanwhile, the results of the current study were in agreement with those of previous studies performed in Brazil [33], in which 1.1% of the population has a hearing impairment, and in Oman, where 55.3 out of 1000 Omani people have hearing impairment [11].

As reported in current research, hearing abilities tend to decline gradually with aging. This is consistent with reports that about one in three people in the United States between the ages of 65 and 74 have hearing loss, and nearly half of the people over the age of 75 have hearing issues [34]. These results also agreed with a previous study that revealed that hearing impairment was positively correlated with age [35].

In the current study, most participants (39.7%) had mild hearing loss. Similar findings were reported in Egypt. In that study, 62.1% of participants had mild hearing loss in the right ear [10]. In Germany, 11.1 million adults have hearing loss, which is primarily mild [31]. Regarding the type of hearing loss, most participants in this study (73.0%) had SNHL, similar to the previous literature that showed that sensorineural hearing loss dominates. A study conducted in Saudi Arabia included 332 cases of hearing impairment, 63.6% of whom had SNHL [36]. High incidences of SNHL could be clarified by the fact that SNHL is associated with several factors, including aging, injury, stroke, disease, exposure to loud noise, high fever, an inherited condition, or specific medications [37,38]. Additionally, children under the age of 12, who are frequently affected by conductive hearing loss due to impacted wax and frequent middle ear infections, were not included in this study’s sample [39,40].

In the literature, finding data about the configuration of hearing loss is difficult. However, in this study, the most common configurations of hearing loss were flat (27.0%) and sloping (15.9%). A study showed that the highest percentage of adult audiometric responses were sloping or u-shaped in configuration. For all configuration categories, the variability of loss at each frequency was higher for children than adults [41]. The present study revealed that bilateral hearing loss (82.5%) was substantially higher than unilateral hearing loss (17.5%). This is similar to the results of studies in Saudi Arabia [36] and Egypt [10].

Additionally, gender differences were not found to affect hearing loss in study participants. The finding contradicts those reported among the Omanis [11], Egyptians [10], and French [42] in which males had a higher risk of hearing loss than females. However, several studies reported that the percentage of females was higher than that of males [31,32,41]. Globally, hearing impairment is higher among males than females [35]. Another study explained that the association between gender and hearing loss is observed as a frequency-specific variation. Thus, men are more impacted by high-frequency hearing loss resulting from conditions such as long-term noise exposure, while women suffer from losing their low-frequency hearing first [43]. In the current study, gender discrepancy was reported in association with the impacted frequency by hearing impairment, as reported by a previous study [43], where males showed more incidences of sloping hearing loss compared to women.

As with the majority of studies, the design of the current study is subject to limitations. First, the sample was 12 years and older; infants or young children were not targeted in the study because these groups have immature auditory systems, and their hearing evaluation requires a different hearing assessment than older individuals. Second, the southern governorates of Jordan were not reached in this study due to their remoteness and extreme primitiveness. It was also difficult to engage people from there in the study because the participants were not cooperative. However, the population of these governorates is relatively small compared to other governorates, as their population is about 766,000 persons. Therefore, to a large extent, the sample is considered representative, and the results could be generalized to similar situations, people, settings, and measures in Jordanian society. Future studies could be run on newborns and children to learn more about the early identification and measurement of hearing loss in Jordan.

## Conclusion

Our study demonstrated the frequency of hearing impairment in the Jordanian population, particularly among those over the age of 18. There were 63 hearing impairments per 1,000 people. This may be less than the regional and international averages. Furthermore, this study also found that most participants’ hearing loss was mild, sensorineural, bilateral, and mainly affected middle-aged people.

## Supporting information

S1 Data (XLSX)
